# inMOTIFin: a lightweight end-to-end simulation software for regulatory sequences

**DOI:** 10.1093/bioinformatics/btag026

**Published:** 2026-01-20

**Authors:** Katalin Ferenc, Lorenzo Martini, Ieva Rauluseviciute, Geir Kjetil Ferkingstad Sandve, Anthony Mathelier

**Affiliations:** Norwegian Centre for Molecular Biosciences and Medicine (NCMBM), Nordic EMBL Partnership, University of Oslo, Oslo 0318, Norway; Norwegian Centre for Molecular Biosciences and Medicine (NCMBM), Nordic EMBL Partnership, University of Oslo, Oslo 0318, Norway; Department of Control and Computer Engineering (DAUIN), Politecnico di Torino, Turin 10129, Italy; Norwegian Centre for Molecular Biosciences and Medicine (NCMBM), Nordic EMBL Partnership, University of Oslo, Oslo 0318, Norway; Department of Informatics, University of Oslo, Oslo, 0373, Norway; Norwegian Centre for Molecular Biosciences and Medicine (NCMBM), Nordic EMBL Partnership, University of Oslo, Oslo 0318, Norway; Department of Medical Genetics, Institute of Clinical Medicine, Oslo University Hospital and University of Oslo, Oslo, 0318, Norway; Bioinformatics in Life Science (BiLS) initiative, Department of Pharmacy, University of Oslo, Oslo, 0316, Norway

## Abstract

**Summary:**

The accurate development, assessment, interpretation, and benchmarking of bioinformatics frameworks for analyzing transcriptional regulatory grammars rely on controlled simulations to validate the underlying methods. However, existing simulators often lack end-to-end flexibility or ease of integration, which limits their practical use. We present inMOTIFin, a lightweight, modular, and user-friendly Python-based software that addresses these gaps by providing versatile and efficient simulation and modification of DNA regulatory sequences. inMOTIFin enables users to simulate or modify regulatory sequences efficiently for the customizable generation of motifs and insertion of motif instances with precise control over their positions, co-occurrences, and spacing, as well as direct modification of real sequences, facilitating a comprehensive evaluation of motif-based methods and interpretation tools. We demonstrate inMOTIFin applications for the assessment of *de novo* motif discovery, the analysis of transcription factor cooperativity, and the support of explainability analyses for deep learning models. inMOTIFin ensures robust and reproducible analyses for studying transcriptional regulatory grammars.

**Availability and implementation:**

inMOTIFin is available at PyPI https://pypi.org/project/inMOTIFin/ and Docker Hub https://hub.docker.com/r/cbgr/inmotifin. Detailed documentation is available at https://inmotifin.readthedocs.io/en/latest/. The code for use case analyses is available at https://bitbucket.org/CBGR/inmotifin_evaluation/src/main/. The version of the code used for this article has been uploaded to Zenodo with DOI: 10.5281/zenodo.17638579.

## 1 Introduction

When developing algorithms or computational models, it is necessary to evaluate their performance in a well-controlled setting (Sandve and Greiff 2022). Although real biological sequences serve as the ultimate test of performance and application, they are often limited in numbers, and one lacks the underlying ground truth they contain. As such, they might not correspond to a gold standard and are often inadequate for thoroughly evaluating a model’s assumptions and behavior in various scenarios, including edge cases.

In the context of regulatory genomics, the goal is to decipher the cis-regulatory code, which governs transcription control through the specific binding of transcription factors (TFs) at binding sites (Zeitlinger *et al.* 2025). Specifically, TFs recognize DNA sequence patterns and act cooperatively at cis-regulatory regions (such as promoters, enhancers, and silencers). Several computational methods have been developed over the years to identify motifs bound by TFs, which are embedded in the DNA sequences of cis-regulatory regions, as well as how their composition regulates transcription. For instance, common tasks consist in discovering TF binding patterns *de novo* within experimentally identified sequences (Van Helden *et al.* 1998), elucidating the cooperativity of binding between pairs of TFs (Rauluseviciute *et al.* 2024), or, more recently, to interpret deep learning models that predict the activity or function of DNA sequences (Sasse *et al.* 2024, Zeitlinger *et al.* 2025). As the underlying ground truth is usually unknown, simulated motifs and DNA sequences are commonly used to assess computational tools during their development and subsequent benchmarking (Tompa *et al.* 2005, Gupta *et al.* 2007, Simcha *et al.* 2012, Shrikumar *et al.* 2019, Rauluseviciute *et al.* 2024). Real biological sequences are often modified to support model evaluation, especially in the case of explainability tools for deep learning models (Gupta *et al.* 2007, Prakash *et al.* 2022).

However, to our knowledge, no single package provides a self-contained, easy-to-use, end-to-end simulation of regulatory sequences by implanting or modifying motif instances (or sites) and grammar in user-provided or simulated background sequences. To address this gap, we developed inMOTIFin, a flexible Python package and command-line tool designed to simulate DNA motifs and background sequences, and to insert motif instances following user-defined grammars. Distinctively, and unlike existing simulators (see Section 1, available as supplementary data at *Bioinformatics* online), inMOTIFin integrates flexible and customizable motif grammars, direct sequence modification capabilities, and a modular architecture, thereby facilitating comprehensive benchmarking and systematic evaluation of regulatory genomics software. Furthermore, inMOTIFin allows for the creation of DNA sequences that contain instances of motifs distributed in groups to simulate cis-regulatory grammars through cooperative binding of TFs. Finally, the package supports the modification of real biological sequences, such as modifying single nucleotides, masking out existing motif sites, or shifting the position of a motif site on a sequence-by-sequence basis. Within inMOTIFin, these simulation tasks are easily parameterized for various use cases. We demonstrate how to utilize inMOTIFin through several use cases: simulating motifs of different lengths and information content, inserting dimers into user-provided or random sequences, and inserting various motif instances in background sequences to mimic biologically relevant scenarios, support the benchmarking of motif discovery tools, and the explainability of deep learning models.

## 2 Features and implementation

### 2.1 Main feature: simulation of sequences with a grammar of TF binding motif instances

**Figure 1:**
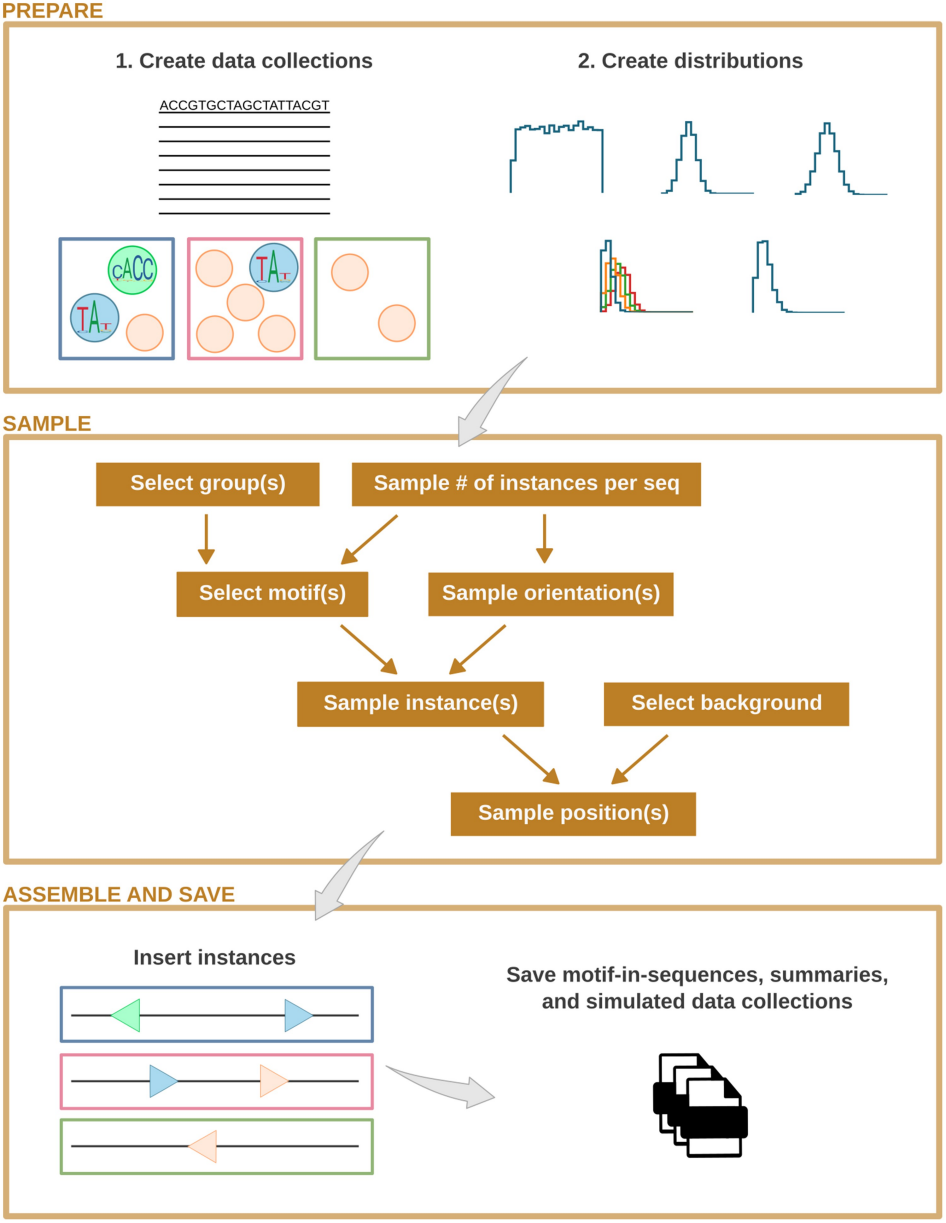
inMOTIFin simulation framework: in preparation, background and motif pools are created, and motifs are assigned to groups. Distributions are set up for sampling of various entities. In the sampling phase, backgrounds, motif groups, and subsequently motifs are sampled. Two motifs are more likely to co-occur in a sequence if assigned to the same group. When motifs from multiple groups can be inserted into the same sequence, the group–group co-occurrence probabilities are conditioned on the previously selected group. Instances are sampled from motifs and inserted into the background sequences in the selected orientations and positions. All newly created data are saved into files, along with additional files to facilitate downstream analyses.

The primary feature of inMOTIFin is the simulation of DNA sequences with motif instances inserted following a grammar of co-occurring motifs (see Fig. 1 and Section 2, available as supplementary data at *Bioinformatics* online). Users can define the motif grammar to specify the spacing between co-occurring motifs (see Section Additional features). Alternatively, for a more flexible grammar, motifs are organized into groups before being inserted into sequences (see Section 3, available as supplementary data at *Bioinformatics* online).

For a simulation task, the user prepares a single configuration file that contains a set of parameters controlling the simulation and sampling of background sequences and motifs, as well as the number, orientation, and positional distribution of motif instances in the final sequences. A comprehensive documentation of the parameters is available at https://inmotifin.readthedocs.io/en/latest/usage/command_line_options.html.

inMOTIFin starts by preparing data collections and distributions to sample from. First, a pool of background sequences and a pool of motifs are imported from user-provided files in standard file formats or simulated from set parameters (see Section Additional features hereafter). Motifs are then organized into groups. Next, the distributions for sampling are set up. Specifically, the probability of selecting a motif is defined as a set of multinomial distributions, which are specified at both the group level (including the occurrence and co-occurrence of groups) and the motif level within groups. The orientation of the inserted motif instance follows a binomial distribution with a user-provided probability of success. The positions of the instances can be either fixed in the middle or sampled from a uniform or a Gaussian distribution. The number of instances per sequence may be a specific user-provided value or drawn from a Poisson distribution. Note that inMOTIFin supports direct user input for each step, where appropriate, allowing, e.g. the modification of real biological sequences with known TF binding motif instances. Following this initial preparation phase, sampling takes place.

During sampling, each component of the simulation is sampled from its respective distribution. To achieve this, inMOTIFin is built on top of DagSim, a simulation framework for causal models (Al Hajj *et al.* 2023). The complete model is represented as a directed acyclic graph with nodes defining random processes or user input (Fig. 1, available as supplementary data at *Bioinformatics* online). The components of the simulation are the selection of groups of expected co-occurring motifs, the choice of motifs, the choice of background sequences, the sampling of motif instances, the sampling of the number of motif instances per sequence, and the sampling of the positions and orientations of the motif instances. The simulated values are passed to their downstream counterparts in each round, resulting in one output sequence at a time. The total number of rounds corresponds to the number of expected sequences.

The package outputs a FASTA file with all sequences with motif instances inserted as well as a corresponding .npz file with the probabilities of each letter in each position. Moreover, complementary output files are also provided to facilitate interoperability with downstream tools. Overall, the output files are:

1. a BED file providing the positions of the inserted motif instances in the sequences;
2. a table in CSV format that includes all the details about the simulated sequences, motifs, instances, orientations, and positions;
3. a JSON file with counts for all sampled entities: backgrounds, groups, motifs, orientations, sequence of motif instances, number of instances per sequence, positions, and motif lengths;
4. a FASTA file providing all background sequences;
5. .npz file with the letter probabilities for each position in the background sequences;
6. a MEME file with the simulated motifs (if applicable);
7. four TSV files detailing the probability of motif-motif co-occurrences produced (if applicable).

Through the Python interface, users have access to more features, most notably better control over the simulation. This includes setting the exact position for each motif instance in each sequence, as well as the option to mask out existing motif sites in DNA sequences with any nucleotide (including Ns). For examples, visit https://inmotifin.readthedocs.io/en/latest/usage/python_module.html.

### 2.2 Additional features

In addition to end-to-end simulation functionality, inMOTIFin enables the specific simulation of random sequences and TF binding motifs. To generate random background sequences, inMOTIFin offers two main options. The user may provide the minimum and maximum length of the sequences (the sequence lengths are sampled uniformly between the minimum and maximum values), the number of sequences, the alphabet, and the frequency of each letter in the alphabet. Based on these parameters, inMOTIFin generates sequences where each position is filled by letters sampled independently and identically distributed (i.i.d.). Alternatively, the user may provide sequences in FASTA format. User-provided sequences can be (i) used as is, (ii) mononucleotide-shuffled, or (iii) used to train a hidden Markov model (HMM) of the desired order from which new sequences will be sampled. For more sophisticated shuffling options, we recommend using the BiasAway or uShuffle tools to create the background sequences and provide them as input to inMOTIFin (Jiang *et al.* 2008, Khan *et al.* 2021).

Similarly to background creation, motifs can either be imported or simulated. Position frequency matrices (PFMs) or position weight matrices (PWMs) in MEME and JASPAR formats are supported for import. Additionally, inMOTIFin integrates *pyJASPAR* (https://doi.org/10.5281/zenodo.4485856), allowing motifs to be automatically retrieved from JASPAR position frequency matrices using their JASPAR matrix IDs (Ovek Baydar *et al.* 2026). When generating TF binding motifs, the parameters are the minimum and maximum length of the motifs (the motif lengths are sampled uniformly between the minimum and maximum lengths), the number of motifs, the alphabet, and the α values for each letter in the Dirichlet distribution. inMOTIFin relies on the Dirichlet distribution to control the average information content per position in the motif (see https://inmotifin.readthedocs.io/en/latest/usage/motif_simulation.html for a detailed explanation and examples). Moreover, inMOTIFin enables the simulation of motifs corresponding to multimers (e.g. a set of fixed spacings between a set of motifs). This can be achieved by providing a set of motifs and multimerization rules including the pairwise distances and the weights of the motifs, useful when motifs are overlapping (see https://inmotifin.readthedocs.io/en/latest/usage/command_line_options.html#multimerisation-of-motifs). The spacings may be negative, allowing for compressed motifs or “noised” motifs when a random signal is combined with a known motif.

### 2.3 Implementation

inMOTIFin is implemented as both a command-line tool and a Python package. The Python package provides access to the individual classes and functions that comprise the simulation framework. The time performance of the simulation modules is *O*(*n*) (see Figs 11–13, available as supplementary data at *Bioinformatics* online). The implementation is modular and well documented, allowing users to utilize polymorphism by defining their classes and functions as replacements for any component (see the complete documentation at https://inmotifin.readthedocs.io/en/latest/usage/python_module_usage.html). Furthermore, inMOTIFin is integrated into the JASPAR 2026 website to support insertion of selected JASPAR motifs into the center of simulated or user-provided background sequences (Ovek Baydar *et al.* 2026).

## 3 Modeling biology

To illustrate the capability of inMOTIFin to generate DNA sequences following a defined, but flexible, grammar of co-occurring motifs, we produced 50 000 random sequences with specific cis-regulatory grammar properties. We aimed to generate sequences that include instances of eight distinct TF binding motifs grouped into three categories for co-occurrence: motifs 0–2 in group 0, motifs 2–5 in group 1, and motifs 1 and 6–7 in group 2 (see Section 3.1, available as supplementary data at *Bioinformatics* online, for detailed probabilities). A pairwise intersection analysis of the sequences containing instances of the eight motifs confirmed that the generated sequences correctly incorporated motif instances according to the specified soft syntax regulatory grammar (Fig. 2, available as supplementary data at *Bioinformatics* online).

inMOTIFin is a rule-based simulation framework, but each step and distribution can be parametrized with user-provided prior information, and sampling introduces variance in the generated outputs. We employed Polygraph (Lal *et al.* 2025), a Python framework evaluating how “real” synthetic DNA elements can be, to illustrate the realism of inMOTIFin-generated sequences compared to sequences generated through FastSeqProp (Linder and Seelig 2021), AdaLead (Sinai *et al.* 2020), and Simulated Annealing (Van Laarhoven and Aarts 1987). Specifically, we used Polygraph’s sequence embedding analysis and its language-modeling queries from HyenaDNA (Nguyen *et al.* 2023). We tested several possibilities of inMOTIFin background generation methods: mononucleotide-shuffling of real sequences, simulation from uniform prior, simulation from GC-rich and GC-poor priors, and HMM-based simulation from real sequences. We additionally considered the generated background sequences with the insertion of a single motif positioned at the center. For the GC-rich simulated sequences, which were most real-like according to HyenaDNA, we also tested the Gaussian insertion of three motifs per sequence (see Section 3.2, available as supplementary data at *Bioinformatics* online, for detailed explanation). The simulated sequences from inMOTIFin, FastSeqProp, AdaLead, and Simulated Annealing were compared with genomic sequences from the human genome. Overall, the analyses show that inMOTIFin sequences are at least “as real as” sequences from the other tools (Fig. 3, available as supplementary data at *Bioinformatics* online). Notably, the GC-enriched sequences show the highest level of likelihood to be “real” as assessed by Polygraph, followed by the GC-poor and Markov-simulated sequences, which retain a higher likelihood than other methods. Finally, inserting a single or multiple motif(s) does not significantly change the likelihood of the sequences to be real (Fig. 4, available as supplementary data at *Bioinformatics* online).

## 4 Use cases

To further demonstrate possible applications of inMOTIFin, we present three illustrative use cases. These examples highlight some of the potential uses of inMOTIFin instead of providing a comprehensive evaluation or benchmarking against the downstream tools used on the simulated DNA sequences.

### 4.1 Use case 1: *de novo* motif discovery

This use case involves inserting instances of simulated TF binding motifs into DNA sequences for application with *de novo* motif discovery tools. In Section 4.1, available as supplementary data at *Bioinformatics* online, we demonstrate how a *de novo* motif discovery tool can be evaluated for its capacity to uncover enriched motifs of varying lengths and information content (IC) generated by inMOTIFin and inserted in random background sequences. By varying the length and IC of simulated motifs, one can determine the optimal detection boundaries of a given tool. In this experiment, we specifically used RSAT’s *de novo* discovery algorithms (Santana-Garcia *et al.* 2022) and compared the extracted motifs with the inserted ground truth motifs using Tomtom (Gupta *et al.* 2007). As expected, long and high IC motifs are consistently identified, while shorter or lower IC motifs are more frequently overlooked by the tool (Fig. 3, available as supplementary data at *Bioinformatics* online). We demonstrate that the lowest threshold for discovery is a length of 6 (corresponding to the user-defined setting of RSAT) and an average IC of 1.4 per position. Shorter motifs can be discovered if the IC is high, and conversely, low IC motifs can be discovered if they are long. This demonstrates how inMOTIFin reliably supports the assessment of *de novo* motif discovery tools, identifying potential limits of tool sensitivity.

### 4.2 Use case 2: generating sequences with co-occurring motifs reflecting pairs of cooperative TFs

To enable complex transcriptional regulation mechanisms, some TFs bind as complexes to cooperatively control transcription (Slattery *et al.* 2014). The inMOTIFin package supports the simulation of multimer motifs and the insertion of groups of motifs within DNA sequences. In this use case, we demonstrate that inMOTIFin can simulate a set of dimer motifs with a user-selected composition of the two individual TF binding motifs that compose the dimers, corresponding to a hard syntax regulatory grammar (see Section 4.2, available as supplementary data at *Bioinformatics* online). We simulated a set of sequence datasets with various dimers inserted in a defined proportion of the complete set of sequences. Anchoring our sequences on the instances of a defined inserted motif, we applied SpaMo (Whitington *et al.* 2011) to identify the secondary motifs that compose the dimers. With sequence simulation, we can test how well these different motif combinations can be found (Fig. 4, available as supplementary data at *Bioinformatics* online). Overall, we observed that the motif identity within the pairs does not have an effect, as all motifs are identified. In contrast, the accuracy of recovery for the specific sites of insertion increases when more motif combinations are present in a single dataset (Fig. 4, available as supplementary data at *Bioinformatics* online). Thus, inMOTIFin robustly facilitates the evaluation of cooperative motifs and motif instance detection under various regulatory complexities.

### 4.3 Use case 3: explainability of deep learning models

The development and use of deep learning models are rapidly growing in genomic research, with an increasing number of frameworks being developed to perform various analyses (Alharbi and Rashid 2022, Zeitlinger *et al.* 2025). Among them, sequence-based models are particularly relevant, where inputs are genomic sequences (Barbadilla-Martínez *et al.* 2025) and models are trained to infer specific functions, particularly pertinent to unravel transcriptional grammar (Zeitlinger *et al.* 2025). In this context, inMOTIFin has the potential to support motif-centered research through easy integration with sequence-based deep learning architectures, thereby enhancing model explainability (Bhagya *et al.* 2023).

A prime example is the input perturbation approaches (Ivanovs *et al.* 2021), which involve selectively modifying an input DNA sequence and measuring the resulting change in the output function. In the case of sequence-based models, inMOTIFin assists in the automated creation of both background sequences (respecting specific conditions, such as %GC content) and their perturbation. Specifically, inMOTIFin allows for the controlled insertion of motif instances to assess changes in the model output. We first show the application of a simple sequence-based model for detecting the GATA and TAL motifs (Section 4.3, available as supplementary data at *Bioinformatics* online). The results demonstrate how the insertion of instances in background sequences alters the model output, highlighting the selective sensitivity of the model to different motifs (Figs 7 and 8, available as supplementary data at *Bioinformatics* online).

Finally, we demonstrate the application of inMOTIFin as a supporting tool to use with *tangermeme* to interpret deep learning models (Section 4.3, available as supplementary data at *Bioinformatics* online) (Schreiber 2025). We illustrate this through two examples.

First, we selected a BPNet model stored in JASPAR 2026 (Avsec *et al.* 2021, Ovek Baydar *et al.* 2026) trained on a ChIP-seq track from the GATA3 TF in MCF-7 cells (BP000597.1). As JASPAR 2026 provides primary and alternative motifs identified by DeepLIFT (Shrikumar *et al.* 2019) and TF-MoDISCo (Shrikumar *et al.* 2018) to be the most contributing to the BPNet model, we systematically assessed the effect of spacing between motif instances of the primary motif and instances of the alternative motifs using *in silico* marginalization (Fig. 9, available as supplementary data at *Bioinformatics* online). As expected, we found that among the identified motifs, the ones activating the most the BPNet model are those that combine GATAA patterns, with pairwise positional preferences (Fig. 9, available as supplementary data at *Bioinformatics* online).

Second, we selected a BPNet model trained on ChIP-seq tracks for the GATA4 TF in HepG2 cells (BP000223.1) to assess the effect of the flexible grammar via grouping of the motifs on the model’s output. Specifically, we constructed all the possible groups of motifs from the seven identified by TF-MoDISCo and stored in JASPAR 2026. Comparing the marginalization of the model’s output between random sequences and the same sequences with motif instances, this analysis revealed that the model gets higher activation when considering the co-occurrence of all the considered motifs (Fig. 10, available as supplementary data at *Bioinformatics* online). Importantly, the results from the inMOTIFin-simulated sequences highlight that while the activation is maximal when considering seven motif instances, it is modulated by the differences of individual motif activities and co-occurrence preferences (Fig. 10, available as supplementary data at *Bioinformatics* online).

In summary, we show that inMOTIFin enables advanced interpretability efforts to elucidate model interpretation and sensitivity.

## 5 Conclusion

Simulation is an important, although time-consuming, part of computational software development. The lightweight, robust, easily integrable, and automated software tool we present here will aid tool evaluation and benchmarking. InMOTIFin is an open-source software built in a modular way to support extensibility, with a focus on the creativity of future users. Beyond the use cases outlined here, there are many other possible applications, including model selection through the simulation of novel types of data based on explicitly stated assumptions; replacement of PWM-based simulation with CWM or seqlet-based (https://tangermeme.readthedocs.io/en/latest/tutorials/Tutorial_A4_Seqlets.html) simulation; or simulation of sequences using an extended alphabet (Viner *et al.* 2024). With inMOTIFin, we aim to pave the way for a more thorough evaluation of models used in the biological context, where ground truth is often lacking and confounding factors can mask signals.

## Supplementary Material

btag026_Supplementary_Data

## Data Availability

The data underlying this article are available in the JASPAR 2026 database at https://jaspar.elixir.no/, and can be accessed with BP000597.1 and BP000223.1. Identical simulated data can be created by running the code at https://bitbucket.org/CBGR/inmotifin_evaluation/src/main/.
