# Supplementary material for: inMOTIFin: a lightweight end-to-end simulation software for regulatory sequences
Source: Bioinformatics. 2026 Jan 20;42(2):btag026. doi: 10.1093/bioinformatics/btag026 (PMC12881827; doi:10.1093/bioinformatics/btag026)
Supplement: btag026_Supplementary_Data [file btag026_supplementary_data.pdf]

Supplementary file for the manuscript inMOTIFin: a lightweight end-to-end simulation software for regulatory sequences

Katalin Ferenc[1], Lorenzo Martini[1,2], Ieva Rauluseviciute[1], Geir Kjetil Sandve[3], and Anthony Mathelier[1,4,5,*]

[1]Norwegian Centre for Molecular Biosciences and Medicine (NCMBM), Nordic EMBL Partnership, University of Oslo, 0318, Oslo, Norway
[2]Department of Control and Computer Engineering (DAUIN), Politecnico di Torino, 10129, Turin, Italy
[3]Department of Informatics, University of Oslo, Oslo, Norway
[4]Department of Medical Genetics, Institute of Clinical Medicine, Oslo University Hospital and University of Oslo, Oslo, Norway
[5]Bioinformatics in Life Science (BiLS) initiative, Department of Pharmacy, University of Oslo, Oslo, Norway

1 Comparison with other tools

Several existing packages support the simulation of motifs, motif instances, background sequences, or the insertion of motif instances into background sequences; their key features are summarized in Supplementary Table 1. Tools such as RSAT, universalmotif, and the JASPAR 2024 web interface implement random motif generation in various ways. Both RSAT and universalmotif allow sampling from user-provided motif instances, while RSAT, universalmotif, and BPNet can generate background sequences. Additionally, RSAT, BPNet, and the discontinued rMotifGen tool support the insertion of motif instances into background sequences. CREME [1], gReLU [2], ML4GLand [3], and tangermeme [4] provide deep-learning model interpretation-specific functionalities embedded with model prediction and evaluation. They are very powerful for marginalizing the effects of sequence modifications and have pre-defined ways to add one or two or remove one motif instance from a given sequence. They often operate on specific motif instances instead of repeated sampling from a given motif pattern. As we show in Supplementary Figures 9-10, they can benefit from the additional flexibility provided by inMOTIFin. inMOTIFin integrates all the regulatory sequence simulation functionalities into a single lightweight and automated solution, as well as providing access to a well-designed backend that stores a meaningful and easily modifiable representation of the components, providing more flexibility and ease of use compared to existing tools.

	**motif and motif instance simulation**	**background simulation**	**insertion of motif instances into background sequences**
JASPAR 2024 [5]	*permute* columns of motifs or *randomize*, which generates motifs similar to the ones in a previous version of the database	NA	NA
RSAT [6]	*random-motif* randomly selects one nucleotide from ACGT for each position, which gets a user-defined value, and the rest are of equal value; *random-sites* samples motif instances from motifs	*random-seq* creates sequences from length, number, and frequencies input; supports Markov-chains from specified organism and various nucleotide sizes	*implant-sites* inserts provided instances into provided backgrounds
universalmotif [7]	*create_motif* from a non-informative Dirichlet distribution and user-provided background frequencies of nucleotides; *shuffle_motifs* shuffles motifs by columns; *sample_sites* samples motif instances from motifs	*create_sequences* creates sequences from length, number, and frequencies input	NA
BPNet [8]	NA	*random_seq* generates a sequence with ACGT bases selected with equal probability for each position	*insert_motif* inserts a single instance of a motif centering at user provided position; *generate_seq* inserts two motif instances, one at the center and one at user provided distances from the centered one

	**motif and motif instance simulation**	**background simulation**	**insertion of motif instances into background sequences**
CREME [1]	NA	random and dinucleotide shuffling	modify the sequences in pre-defined ways, such as context swapping or repeating regions
gReLU [2]	*motifs_to_strings* function is defined that can sample an instance from a motif	randomizing sequences (not necessarily backgrounds) with functions such as using reverse complement, shifting and jittering, and *in silico* mutagenesis	add motif instances and runs tests in pre-defined ways, such as evaluating the effect of distance between two motif instances or keep the motif instances in place and shuffle the backgrounds
tangermeme [4]	NA	creates random one-hot encode ACGT sequence parametrized by letter probabilities, single and dinucleotide shuffle of input sequences, reverse complementation, *in silico* mutagenesis	insert one or multiple motif instances into defined positions and optionally with relative positions to each other

	**motif and motif instance simulation**	**background simulation**	**insertion of motif instances into background sequences**
ML4GLand [3]	NA	SeqPro can generate random sequences with independent, identically, and equally distributed letters from DNA or protein alphabet; or it can shuffle, jitter, or reverse complement a sequence. SeqExplainer can also shuffle the input sequences	SeqExplainer can add motif instances in pre-defined positions. EUGENe's interface with SeqExplainer allows the addition of two motifs: one in the middle, the other in the background at various distances defined by the user
rMotifGen* [9]	NA	NA	user can provide nucleotide frequencies and motifs, the output is a random sequence with motif instances and their locations
inMOTIFin [this publication]	creates random motifs based on length range and alpha values for Dirichlet prior from which motifs are generated, creates multimers from motifs and pairwise distances, samples instances from motifs	creates sequences from provided length range, number, and letter probabilities, or fit a hidden Markov model to sample new sequences	single parametrized framework to connect all tasks and allow control for selecting number of motifs and instances, co-occurrence frequency of motifs, positions and orientations to build the final sequence; additionally, all simulated motifs and sequences, as well as imported sequences, are assigned position-specific letter probabilities

Supplementary Table 1: **Comparison of features with other simulation tools**. *Assessed from the manuscript, because the code and GUI are not available anymore.

2 Sampling procedure

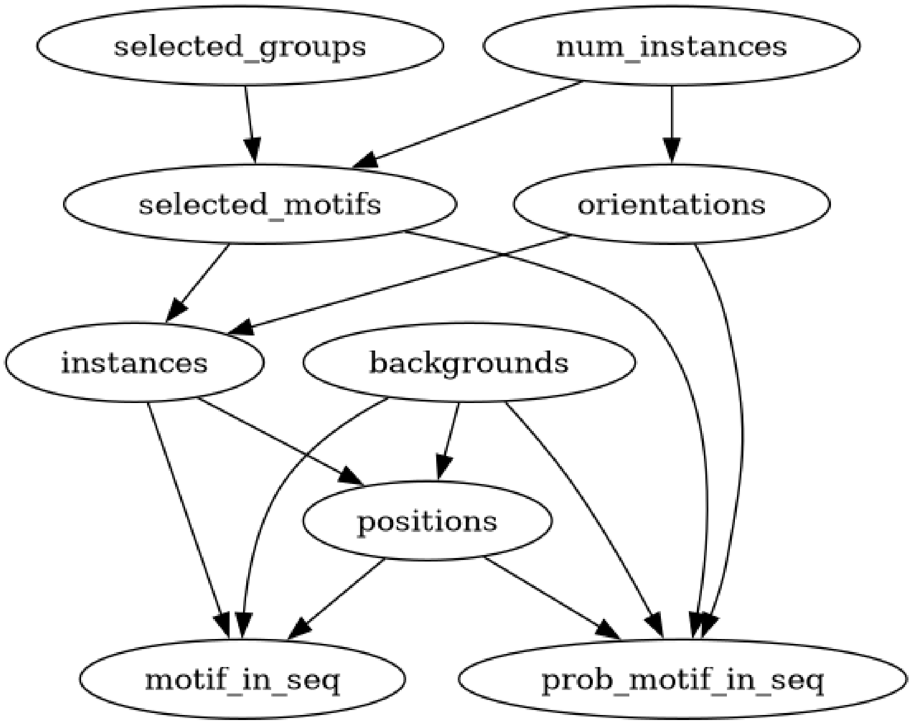

Supplementary Figure 1: **Overview of the directed acyclic graph supporting the simulation framework in inMOTIFin.**

The complete direct acyclic graph supporting the simulation framework implemented in DAGSim [10] for inMOTIFin is illustrated in Supplementary Figure 1. The number of motif instances (*num_instances*) to be inserted into the background sequences can be set as a single integer or can be sampled from a Poisson distribution. The motif position probability matrices are selected with replacement from a list of motifs (*selected_motifs*), which can be read from a file, accessed directly from the JASPAR database [5] using the pyJASPAR package [11], or sampled from a user-defined Dirichlet distribution. The orientations (*orientation*) are sampled from a binomial distribution. Instances (*instances*) are sampled from the motif on the fly, given the alphabet of the sequences. The background sequences (*background_id*) are sampled, either from a list of sequences provided by the user or generated by sampling from a distribution where the user provides the probability of each letter. The positions (*positions*) where the motif instances are inserted within the background sequences are centered or sampled from a uniform or a Gaussian distribution. The final sequence (*motif_in_seq*) is assembled using the selected background sequence, the selected motif instances, and their selected positions and orientations. Beside the final sequences, inMOTIFin also

provides a probabilistic representation of the output (*prob_motif_in_seq*) representing the letter probabilities for each position. Motifs are organized into groups, allowing groups of motifs to be preferentially selected together (*selected_groups*) based on predefined or simulated group frequencies and transition probabilities.

3 Modeling biology

3.1 Grammar of motif co-occurrence simulation

The inMOTIFin tool enables motifs to be organized into groups, each with its own defined probability of being selected. Within each group, the motifs are selected according to the probabilities specified. Additionally, users can define probabilities for the co-occurrence of different motif groups. Here, we illustrate how this setup enables precise control and insertion of co-occurring motifs into sequences.

Four files are provided for this simulation and contain the following:

- the probabilities of selecting each group for insertion (Table 2).
- the conditional probabilities for selecting additional groups after a previous group has been chosen, applicable when sequences contain multiple groups (Table 3). For this specific simulation, ignoring this file, each sequence contains only one group.
- the assignment of individual motifs to their respective groups (Table 4).
- the probabilities of selecting specific motifs within each group (Table 5), which determines the co-occurrence frequency of motif pairs.

Further details can be found at `https://inmotifin.readthedocs.io/en/latest/usage/command_line_options.html`.

The assignment of the underlying groups making up the cis-regulatory grammar was assessed using Intervene [12] to confirm the co-localization of motif instances between within and between groups (Supplementary Figure 2).

Group Name	Probability
group_0	0.2
group_1	0.3
group_2	0.5

Supplementary Table 2: **Motif group probability values**. This is similar to the example file *group_freq_file.tsv* available in the documentation.

	group_0	group_1	group_2
group_0	1	0	0
group_1	0	1	0
group_1	0	0	1

Supplementary Table 3: **Group pair probabilities**. This is similar to the example file *group_group_file.tsv* available in the documentation.

Group Name	Motifs
group_0	motif_0, motif_1
group_1	motif_2, motif_3, motif_4, motif_5
group_2	motif_6, motif_7, motif_1

Supplementary Table 4: **Motif assignments to the groups**. This is similar to the example file *group_motif_assignment_file.tsv* available in the documentation.

	group_0	group_1	group_2
motif_0	0.6	0	0.1
motif_1	0.4	0	0
motif_2	0	0.25	0
motif_3	0	0.25	0
motif_4	0	0.25	0
motif_5	0	0.25	0
motif_6	0	0	0.3
motif_7	0	0	0.6

Supplementary Table 5: **Motif probabilities within the groups**. This is similar to the example files *called motif_freq_file.tsv* available in the documentation.

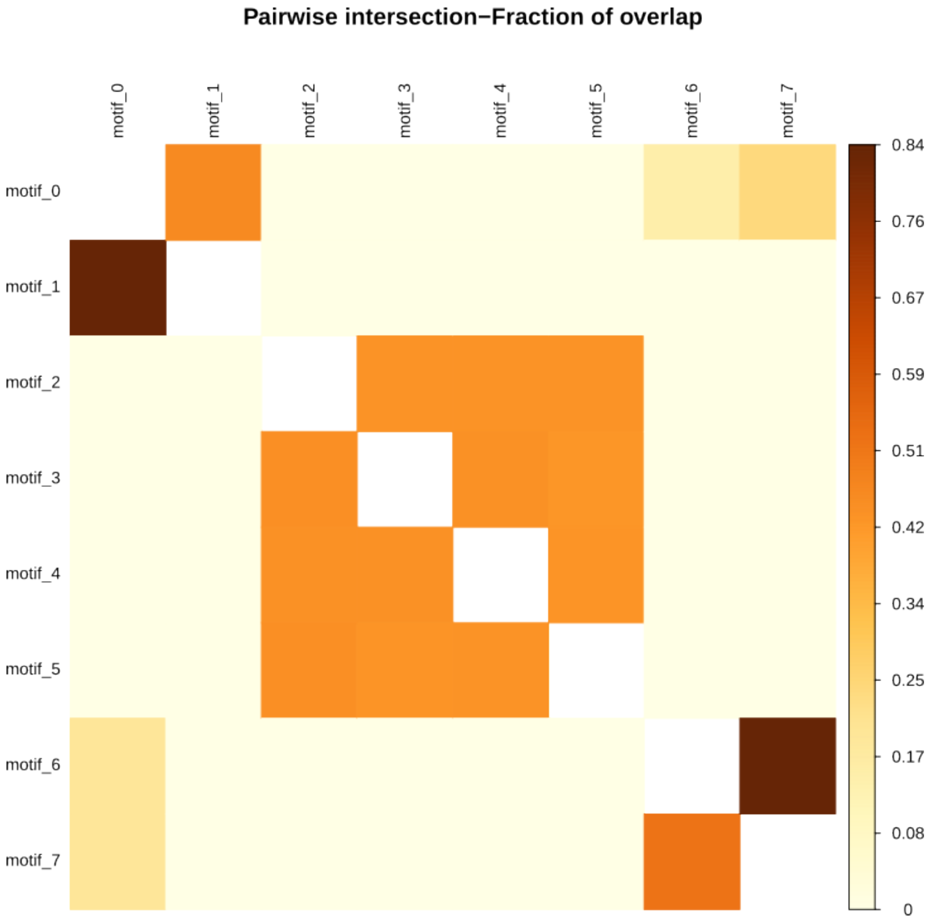

Supplementary Figure 2: **Pairwise occurrences of inserted motifs within the simulated sequences**. The heatmap provides the frequencies of co-occurrence of the motif instances within the simulated sequences, confirming the regulatory grammar of the groups provided as input to inMOTIFin.

3.2 Polygraph analysis

Polygraph [13] is a Python framework for evaluating synthetic DNA elements. We used it to assess the realism of inMOTIFin's background sequence generation methods and to study the effects of simple motif insertion. First, we generated 1,000 200-bp sequences using the main background generation methods provided by inMOTIFin. Namely, these are: (i) reading from a fasta file real genomic sequences (Real); (ii) randomly shuffling real sequences (shuffle); (iii) simulating *de novo* sequences by randomly selecting nucleotides with a uniform prior (simulated) or (iv) from a GC-biased prior, either (A: 15%, C: 35%, G: 35%, T: 15%) to obtain GC-rich sequences (GC-rich), or (v) (A: 30%, C: 20%, G: 20%, T: 30%) to obtain GC-poor sequences (GC-poor); (vi) simulating *de novo* sequences by sampling nucleotides from a fitted HMM (markov). To provide a reference, all inMOTIFin results were compared with Polygraph's own benchmark sequences, which include random sequences (random), native genomic sequences (native), and synthetic sequences generated using three methods: FastSeqProp [14], AdaLead [15], and Simulated Annealing [16].

We focused on two primary analyses from Polygraph. First, the sequence embedding analysis, where each sequence is represented by its k-mer (with $k = 5$) composition and visualised via dimensionality reduction to evaluate sequence similarity. Second, the LLM-based likelihood analysis, which uses HyenaDNA [17], a genomic foundation model pretrained on the human reference genome. By querying the LLM, Polygraph estimates the likelihood of each sequence, which is a proxy for its realism relative to the human genome.

Supplementary Figure 3 shows the UMAP representation of the sequence embeddings. Notably, the HMM-simulated and GC-poor sequences appear most similar to the real sequences. In contrast, the GC-rich sequences form a separate group, as do the synthetic sequences from Polygraph. Interestingly, the results from the LLM-based analysis are somewhat different (Supplementary Figure 3). As expected, completely random sequences have the lowest likelihood. However, among the inMOTIFin-generated sequences, the GC-rich sequences show the highest likelihood, comparable to real sequences, followed by GC-poor and HMM-simulated sequences. Moreover, inMOTIFin's sequences generally exhibit higher likelihood scores than those produced by Polygraph's reference methods, further confirming the robustness of inMOTIFin's generation process despite its rule-based nature.

Beyond background sequences, we assessed the effect of motif insertion. We tested, across all background types, the insertion of a single motif in the centre to assess the impact of a slight sequence change. The inserted motif is randomly selected from a group of five JASPAR motifs (CTCF, MAX, YY1, MAZ, and ZNF143). Additionally, for the GC-rich background sequences, which were closest to real sequences according to the Polygraph analysis, we tested a stronger modification by testing the Gaussian insertion of three motifs per sequence, each randomly selected from one of three motif groups. The likelihood after insertion only slightly increases (Supplementary Figure 4).

4 Extended results

4.1 Use case 1: *de novo* motif discovery

The following parameters of inMOTIFin were used in this experiment. The motif lengths were set between 4 and 9, with an average information content (IC) per position ranging from 0.1 to 1.8. Ten motifs were created for each setting. We simulated 1000 random background sequences of length 100 with equal and independent probabilities of sampling each base at each position. In each simulation round, 50,000 sequences were sampled with replacement from the 1,000 randomly generated sequences. One motif instance was inserted in 95% of these sequences. The location of the motif instances was uniformly distributed across the sequence. Each instance had a forward orientation. Each simulation round was repeated 10 times with different random seeds. On average, each motif was observed 4750 times.

To discover the injected motifs, RSAT was used with the following parameters. We applied the following *de novo* discovery algorithms: oligos, positions, and local_words, with an oligo length of 6 to 7. In each round, the number of motifs was set to 10, matching the known ground truth. Thus, we evaluated the boundaries of discovery with slightly shorter and longer motifs than expected by the discovery algorithm.

In the comparison step, all motifs from the 10 rounds of each setting were pooled together. This created a pool of 100 motifs. For each round, the 10 discovered motifs were compared with the pool using Tomtom. True positive motifs were those that were discovered and matched with the ground truth using Tomtom with an E-value threshold of 0.05. False positives were motifs that were inserted but not found after motif comparison between the discovered and the ground truth. False negatives were the motifs that matched some motif from the pool that was not inserted in the specific round. True negatives were the motifs that were not found and indeed not inserted.

The results are shown in Supplementary Figure 5. Each circle is colored by the average Matthews Correlation Coefficient (MCC) calculated across simulation rounds. We can observe that longer motifs are easier to discover, even beyond the expected maximum length. However, even shorter nucleotide motifs as a minimum expectation are not found. This is expected as the discovery algorithm sets a seed for each motif that can grow but not shrink. Similarly, the higher average IC per position increased the probability of discovering the motif. Interestingly, as low as 0.5 average IC per position was sufficient to find motifs when their length was at least 8.

4.2 Use case 2: generating sequences with co-occurring motifs reflecting TF cooperativity

Several tools are available for discovering co-occurring or dimeric TF binding motifs. For instance, SpaMo identifies potential cooperative TFs within genomic regions [18]. To perform this analysis, SpaMo requires a set of input sequences, each containing a primary motif positioned approximately at the center of the sequences. The software then searches upstream and downstream of the anchor motif to identify secondary motifs that are significantly enriched.

To simulate the input for this tool, we used inMOTIFin to create four dimeric motifs, where one part was identical in all (referred to as a primary motif). We chose the CTCF motif from the JASPAR database [5] (motif ID MA0139.2). Four other motifs, MAX (MA0058.4), YY1 (MA0095.4), MAZ (MA1522.2), and ZNF143 (MA0088.2), were chosen to represent the second motif of the dimer motifs. We simulated random sequences with four dimers inserted with different proportions in the same number of sequences (6000 sequences, each case simulated ten times). The proportions ranged from 0 to 1, where some dimers were absent or inserted in all sequences. The goal was to observe whether SpaMo could identify the secondary motif in the sites where it was inserted and how varying the proportions of the secondary motifs affects their discovery. We repeated each insertion case 10 times and summarized using the MCC (Supplementary Figure 6).

The results in Supplementary Figure 6 show that all the motifs were significantly enriched in all cases. The identity of the secondary motif does not affect their recovery. However, there is a variation in MCC scores. The average MCC scores for 10 repeats were the highest when all four dimers were present in the set of sequences and lowest when only one dimer was inserted. Notably, SpaMo has a high motif mismatch rate. The algorithm searches for enriched motifs using a collection of known motifs (in this case, we used JASPAR 2024 non-redundant CORE collection) [5]. Therefore, multiple motifs representing binding of TFs from the same family will be found enriched.

Overall, with inMOTIFin sequence simulation, we can systematically evaluate motif enrichment tools by creating sequences with a variety of dimer motifs present. Our analysis revealed that a tool can exhibit different performance depending on the motifs present and their frequency. This evaluation would be difficult, if not impossible, with real data.

4.3 Use case 3: tool for explainability of deep learning models

We demonstrate the application of inMOTIFin for input perturbation analysis of a simple deep-learning (DL) model. This model accepts 200 bp sequences as input and identifies the presence of GATA motifs, TAL motifs, or both through a final output layer consisting of three neurons. We compare the model outputs between original background sequences and identical sequences with motifs inserted. Specifically, 1000 background sequences (200 bp each) were generated by shuffling real genomic sequences. Motif insertion was then performed by inMOTIFin, producing three distinct sequence sets: sequences containing only GATA motifs, sequences containing only TAL motifs, and sequences containing both GATA and TAL motifs. TF binding motifs were directly obtained from the JASPAR 2024 non-redundant CORE collection (motif IDs MA0037.3 and MA0091.2, respectively).

For sequences containing a single motif type, sampled motif instances were inserted precisely at the center of each sequence. For sequences containing both motifs, the two instances were inserted at uniformly distributed positions. Consequently, we generated four sets of 1000 sequences: three motifs inserted in each background. These were subsequently classified by the model. For each sequence, the model output was recorded as activation levels of the three output neurons. We analyzed the differences in activation levels between each background sequence and its corresponding motif-

inserted versions. Figure 7 plots the average difference in activation across the 1000 sequences for each motif-inserted set, clearly illustrating how motif insertion alters the activation of the respective output neuron.

Variations in activation among the different motif-inserted sets reflect characteristics of the original model's architecture and training conditions. Notably, the TAL motif used in this analysis may differ slightly from the motif version used initially during model training, which accounts for the lower activation increase in the TAL-specific neuron. Such analyses are valuable for understanding how the presence or absence of specific motifs impacts model predictions, thereby offering insights into underlying biological mechanisms.

Other approaches to DL explainability include gradient-based attribution methods (e.g., SHAP [19] or DeepLift [20]), which map the contributions to the output for each input feature. For an input sequence, this means mapping them to each nucleotide. Analogously to the previous example, inMOTIFin assists in the creation of *ad-hoc* sequences with inserted motifs to assess the contribution of nucleotides in motif instances to the model output. This type of analysis helps highlight the base-specific importance score and is naturally comparable with motifs' PWM. Figure 8 plots the scores obtained on a sequence with both motifs inserted, with respect to the three output neurons. The blue box highlights the GATA instance, and the green box highlights the TAL one. The coordinates are easily obtained from the inMOTIFin-generated BED file. The plots illustrate how the three neurons selectively recognize the bases associated with the two motifs. The TAL instance probably does not entirely match what it was trained on, as previously discussed. Again, inMOTIFin is a valuable tool for creating *ad hoc* simulated sequences, which helps in understanding the model's behavior.

Beyond the toy example shown above, we conducted two experiments using two BPNet models stored in the JASPAR 2026 database [8, 21]. First, we investigated the effect of spacing between most contributing motifs identified for the BPNet model's accuracy. Specifically, we selected the BP000597.1 model, which was trained on ChIP-seq data for the GATA3 TF in MCF-7 cells. We considered the primary motif as stored in JASPAR 2026 with 15 other motif patterns identified for insertion into random sequences with inMOTIFin. To construct the background sequences, we downloaded ATAC-seq data from the MCF-7 cell line from GEO (GSE273908 [22]) and trained an HMM of order one on 2,500 sequences from this dataset. From the trained HMM model, we sampled 100 sequences and used these as background sequences for motif insertions. Next, we created dimers of the primary motif and each of the other motifs with different spacing (from 0 to 9 bp). Finally, we inserted sampled instances at random orientations and positions within a 600 bp window around the centre of each sequence. This process was repeated 100 times for each background sequence, and the marginalised activity of the model was obtained using *tangermeme* and averaged across runs. As expected, the most activating partners are those containing a GATA motif (Supplementary Figure 9). Furthermore, a preference for specific distances for some pairs can be observed. For example, the primary motif paired with motif_13 shows high activation when they are no more than four nucleotides apart, with a preference for 0-1 bp. In comparison, motif_6 activates the most when it is five nucleotides apart from the primary motif.

Next, we assessed the effect of a flexible grammar with co-occurrence of motifs at

non-predefined distances. We selected the BP000223.1 model stored in JASPAR 2026 and trained on the GATA4 TF ChIP-seq data from HepG2 cells. Using inMOTIFin, we created GC-rich (60% GC) i.i.d. background sequences. We downloaded the primary motif, along with the six other motif patterns identified by TF-MoDISCo [23] as most contributing to the model's accuracy. From this set of seven motif patterns, we created all combinations of 1, 2, 3, and 4 motifs, with and without the primary motif. As a control, we created 15 motifs with matching %GC and information content and assigned these to three groups, from which we randomly sampled 1, 2, 3, and 4 motifs without replacement. We observed that the marginalisation values increase with the number of motifs considered in a group (Supplementary Figure 10).

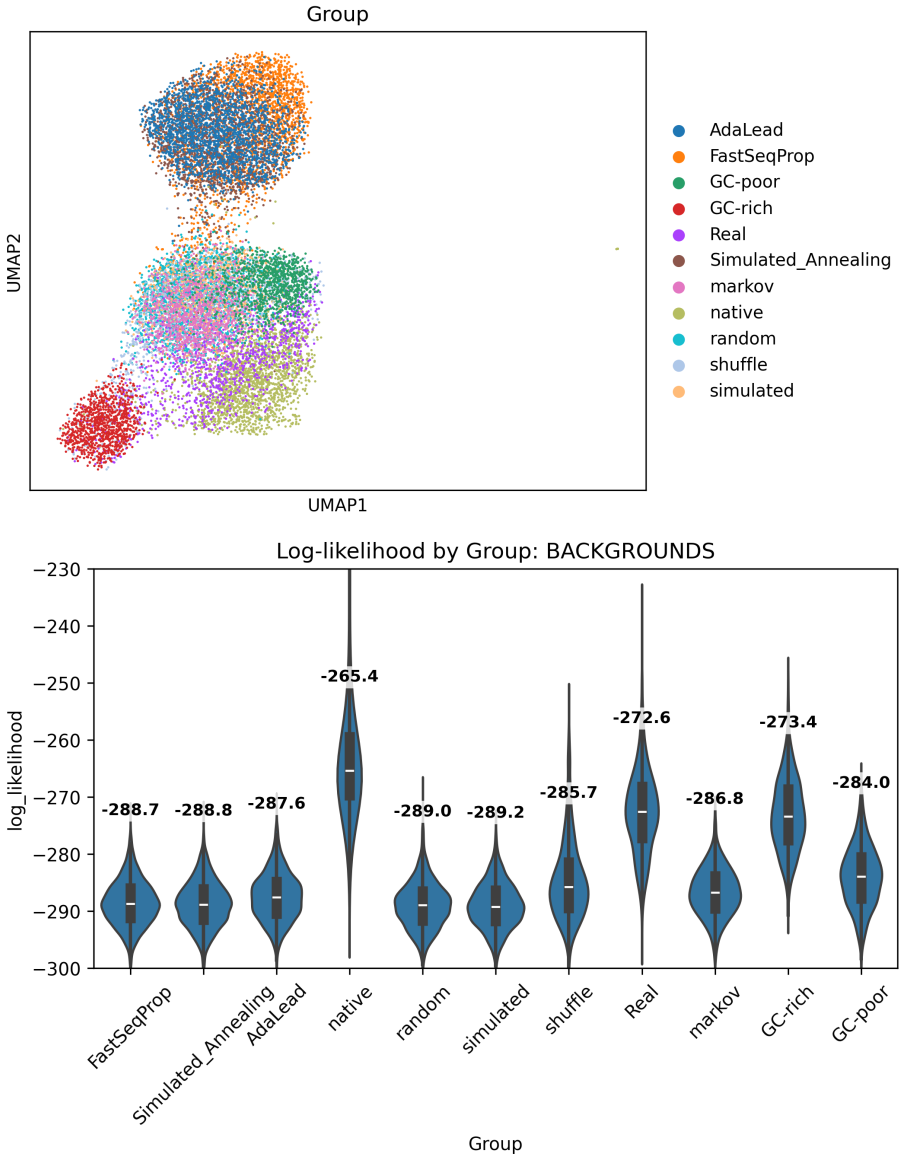

Supplementary Figure 3: **Polygraph analysis on background sequences. Top.**The UMAP visualization of sequences embedding shows clear groups, with the inMOTIFin generated sequences nearer to the real ones than other methods. **Bottom.** The likelihood from HyenaDNA LLM, shows that the inMOTIFin generated sequences have generally higher likelihood than the Polygraph reference sequences. In particular GC-rich sequences have a likelihood comparable with real ones.

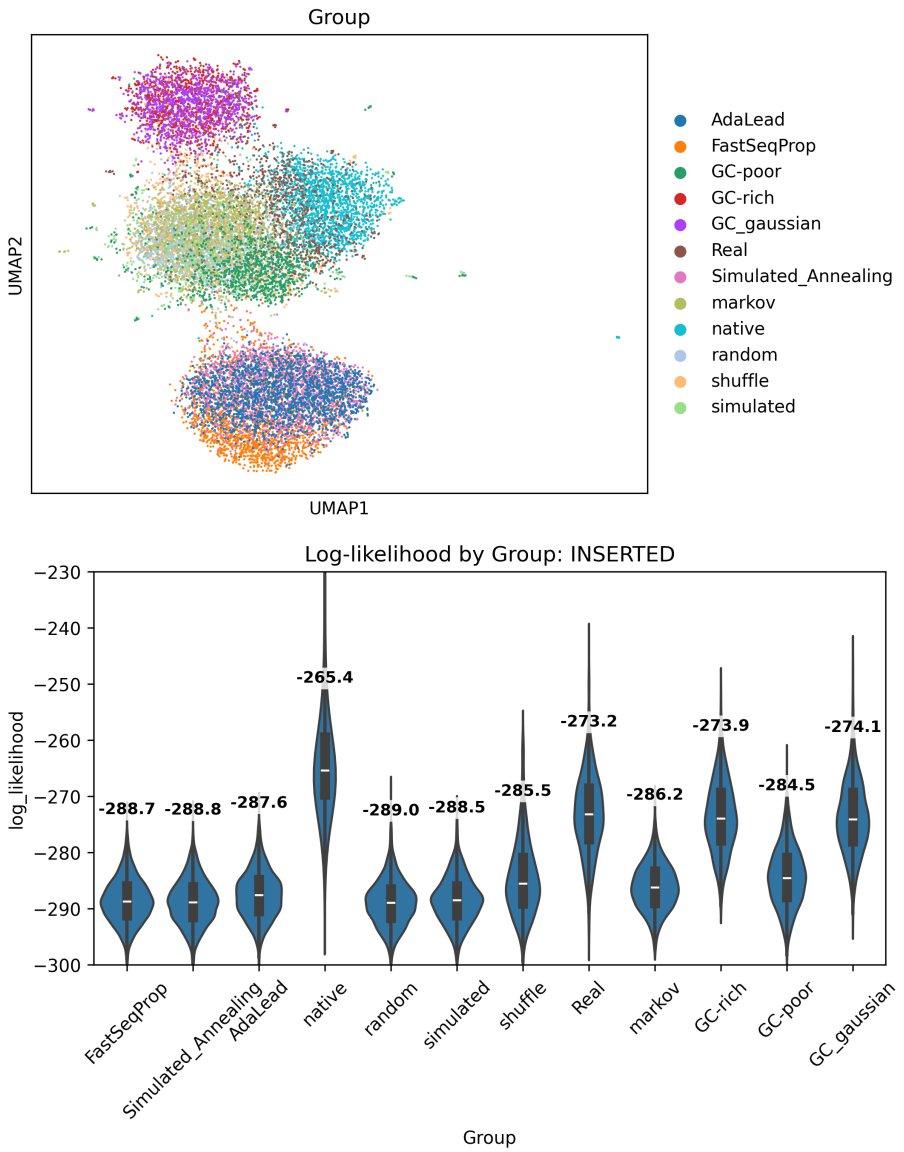

Supplementary Figure 4: **Polygraph analysis on motif-inserted sequences. Top:** The UMAP visualization does not change much with the addition of inserted motif instances, except the presence of outliers. **Bottom:** The likelihood from HyenaDNA LLM, shows that the the motif insertion does not change much the overall likelihood, even increasing it slightly in most cases. Even the gaussian insertion of multiple motifs (so changing an higher portion of the sequences) does not affect much the final likelihood, showing the reliability of inMOTIFin simulation to generate sequences.

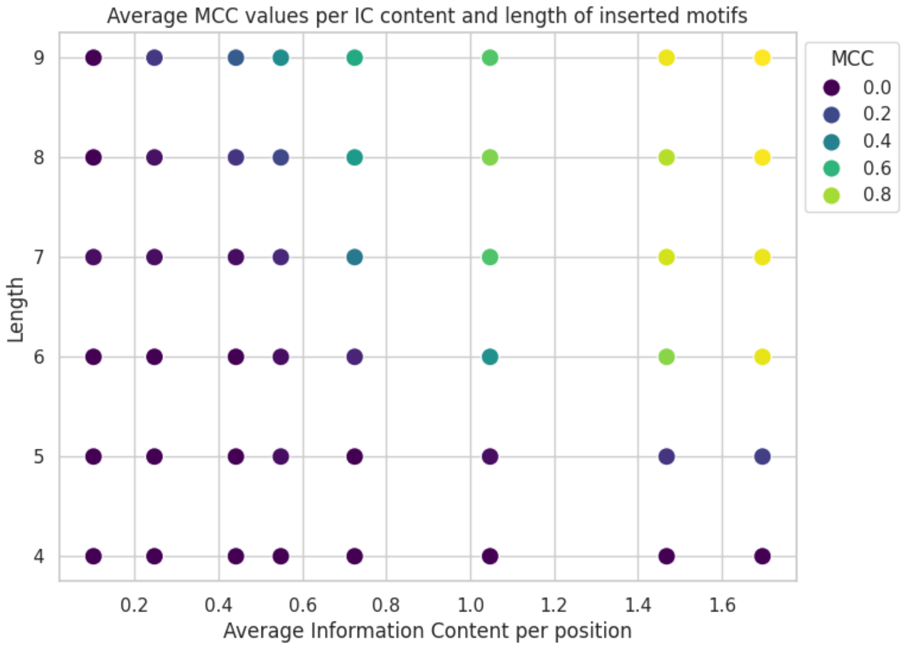

Supplementary Figure 5: **Effect of IC content and length on the *de novo* motif discovery with RSAT.**

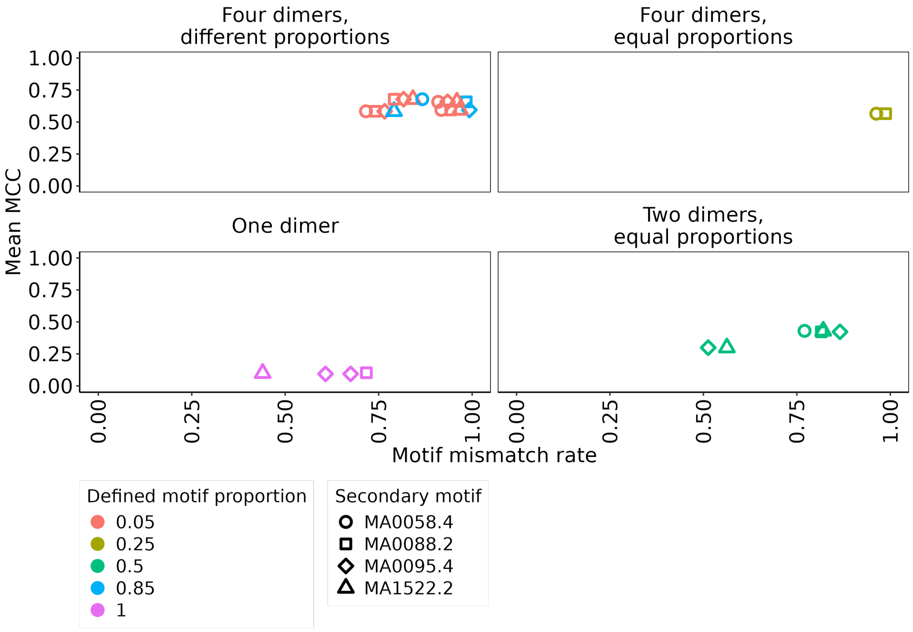

Supplementary Figure 6: **Searching for secondary TF motifs from simulated sequences with inserted primary-secondary motif dimers**. The motif identity does not significantly influence the enrichment analysis results, whereas the presence of different dimers does. The example tool used in this analysis consistently identifies the target motifs as enriched, but recovers their instances more effectively when four dimers are inserted and less effectively when only one dimer is present.

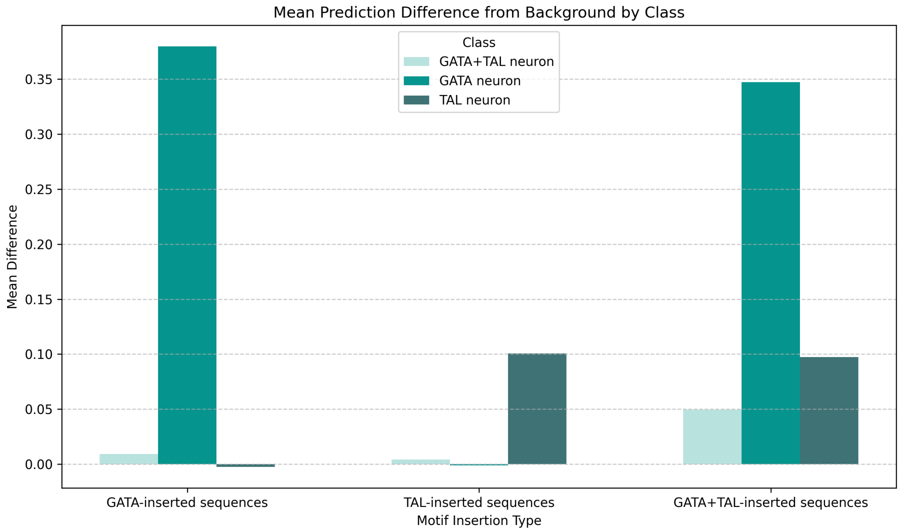

Supplementary Figure 7: **Input perturbation of DL model, by motif insertion.** The insertion of GATA and/or TAL instances affects the output neurons with respect to the same background sequences.

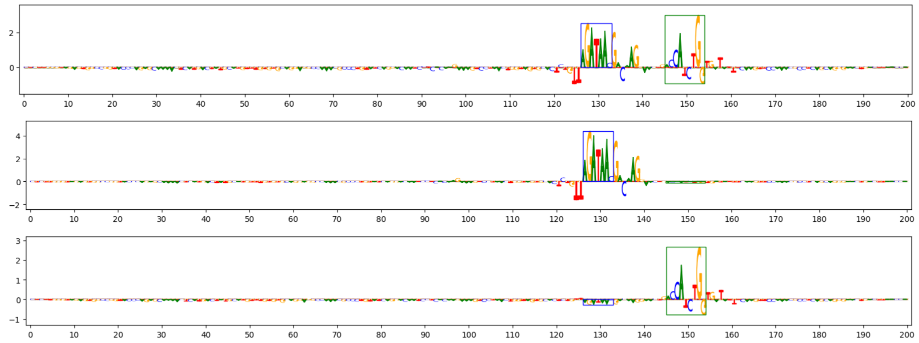

Supplementary Figure 8: **Overview of DeepLift contribution scores**. The scores represent the results of the calculation performed on a sequence containing both GATA (blue box) and TAL (green box) motif instances. The calculation is performed on the GATA+TAL, GATA, and TAL neurons, respectively.

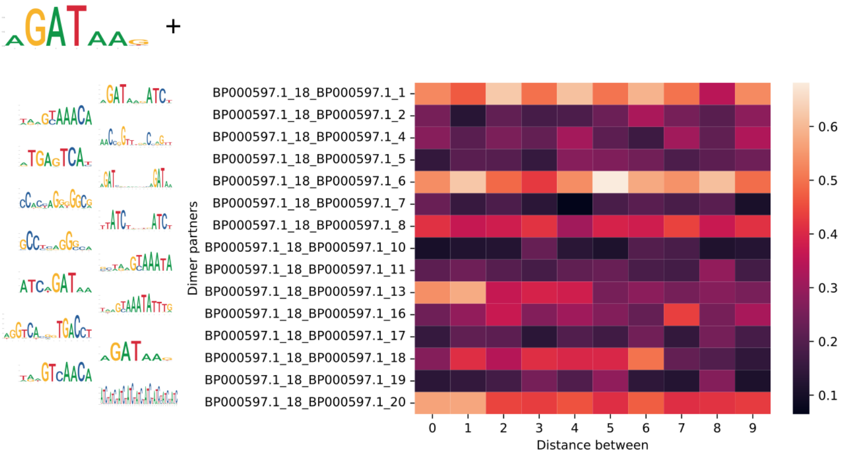

Supplementary Figure 9: **Activation of BPNet model trained on GATA3 ChIP-seq data with dimers of various motifs**. Each row is a motif pair, and each column is a specified spacing between the two elements. The color scale shows the average of BPNet model's activation relative to the 100 different backgrounds. Activation was calculated across pairs of background and insertion into the selected background.

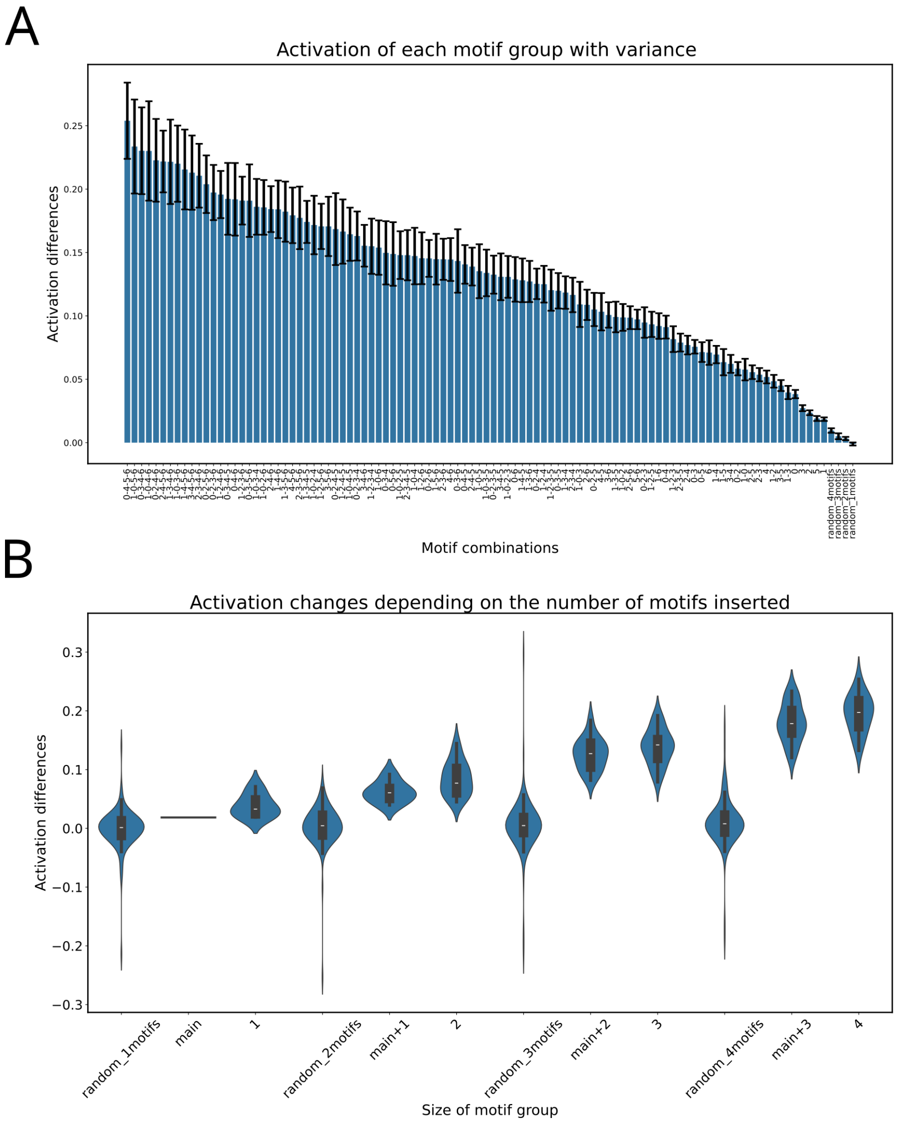

Supplementary Figure 10: **Activation of the BPNet model trained on GATA4 ChIP-seq data with groups of 1-4 motifs. A)** means and variances of activation difference (i.e. marginalisation values) between background sequences and background sequences with specified groups of inserted motifs. Each number represents a motif; motif 0 is the primary motif identified in JASPAR 2026. As a control, 1, 2, 3, and 4 random, GC- and information content-matched motifs were inserted to the same set of background sequences. **B)** The activation differences grouped by number of motifs in each group, and whether the primary motif is present or the motifs are random ones.

5 Performance

The evaluation was performed on a desktop machine with an Intel® Core™ i7-10750H × 12 processor. Supplementary Figures 11-13 illustrate the outcome of this test. The time complexity of each simulation type is O(N).

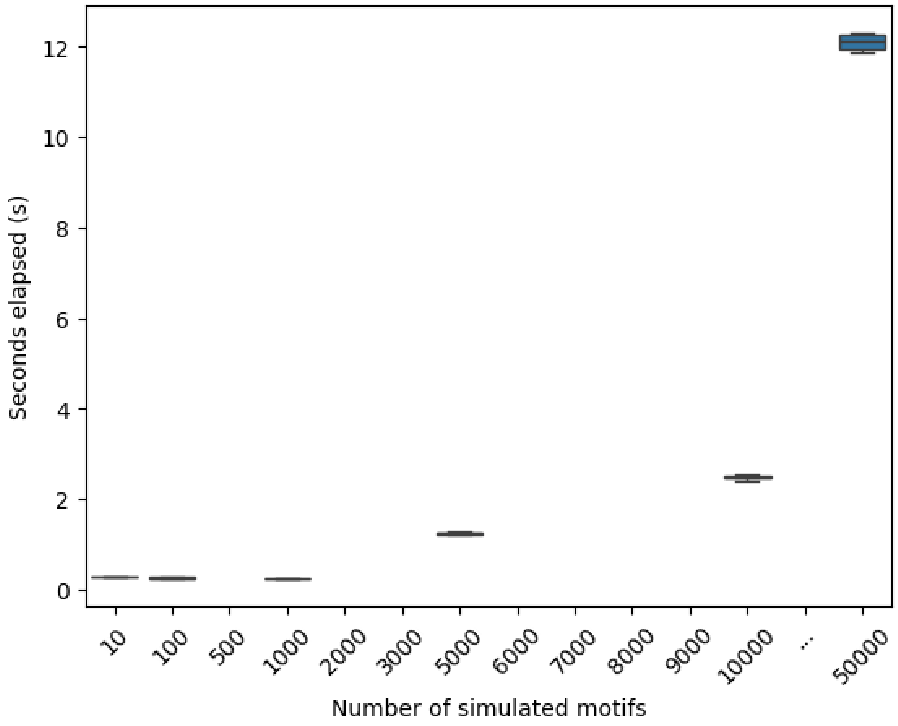

Supplementary Figure 11: **Performance of motif simulation**. An increasing number of motifs of length within the range of 5 to 9 have been simulated.

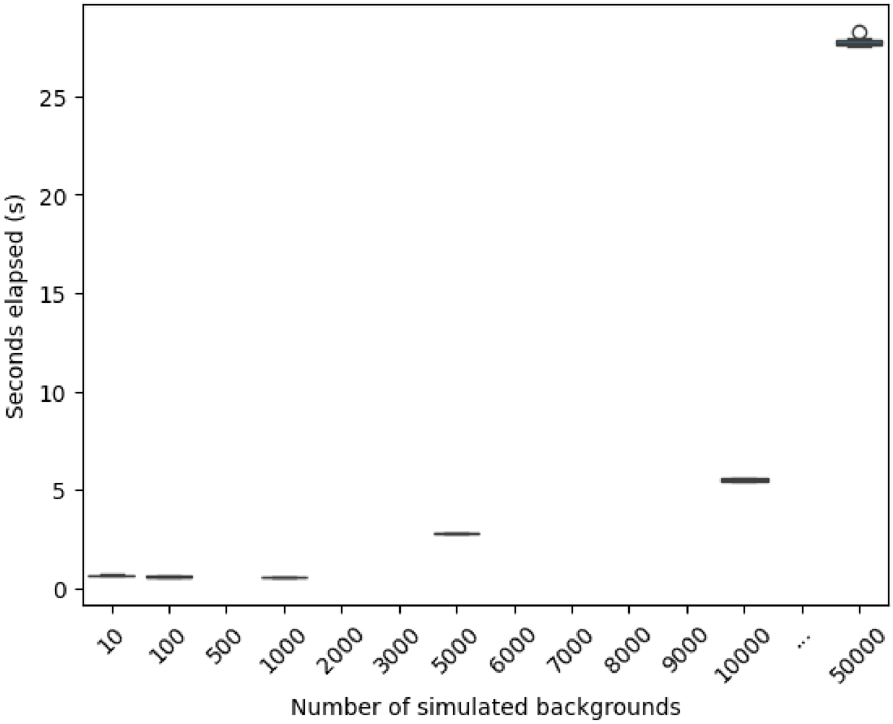

Supplementary Figure 12: **Performance of background simulation.** An increasing number of background sequences of length 25 have been simulated.

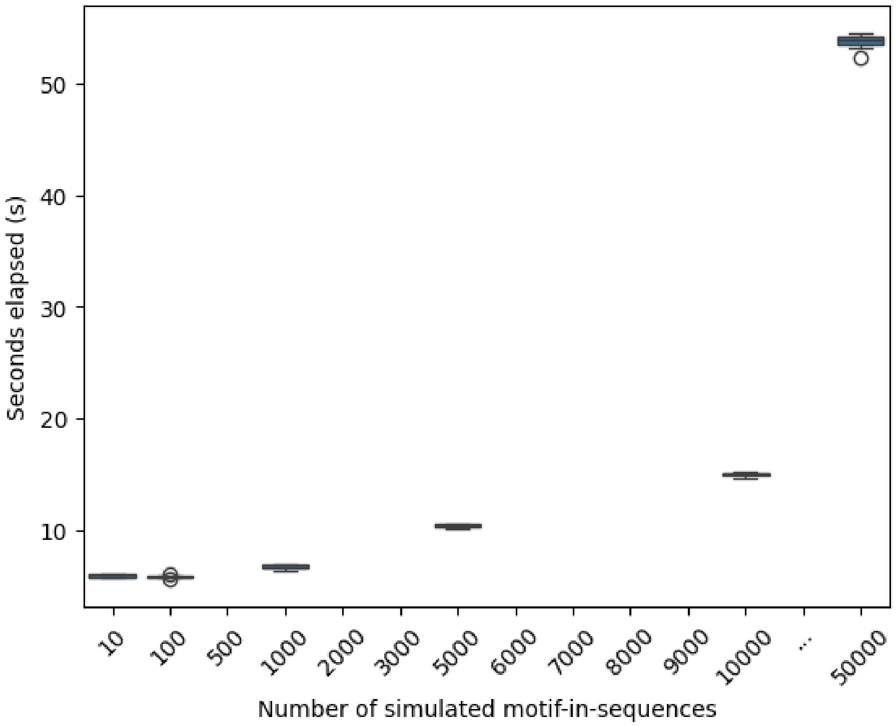

Supplementary Figure 13: **Performance of motif-in-sequence simulation**. In the preparation phase, 50 motifs of lengths 5-9 were simulated and assigned to 5 groups, with a maximum size of 10 (average size 7). The probability of group selection was assigned randomly, allowing differences up to 8-fold. Within the groups, the probability of selection was equal for all motifs. Furthermore, 1000 backgrounds of length 250 were simulated. In the sampling phase, three groups and five motifs were selected per sequence. The positions of inserted motif instances were sampled from a Gaussian mixture model consisting of two distributions, centered at 50 and 210 bp, with variances of 10 and 3, respectively. The probability of motif instance orientation was 60% forward and 40% reverse.

References

[1] Shushan Toneyan and Peter K Koo. Interpreting cis-regulatory interactions from large-scale deep neural networks. *Nature Genetics*, pages 1–11, 2024.

[2] Avantika Lal, Laura Gunsalus, Surag Nair, Tommaso Biancalani, and Gokcen Eraslan. gReLU: A comprehensive framework for DNA sequence modeling and design, 2024.

[3] Adam Klie, David Laub, James V. Talwar, Hayden Stites, Tobias Jores, Joe J. Solvason, Emma K. Farley, and Hannah Carter. Predictive analyses of regulatory sequences with EUGENe. *Nature Computational Science*, 3(11):946–956, 2023.

[4] Jacob Schreiber. tangermeme: A toolkit for understanding cis-regulatory logic using deep learning models, 2025.

[5] Ieva Rauluseviciute, Rafael Riudavets-Puig, Romain Blanc-Mathieu, Jaime.A Castro-Mondragon, Katalin Ferenc, Vipin Kumar, Roza Berhanu Lemma, Jérémy Lucas, Jeanne Chèneby, Damir Baranasic, Aziz Khan, Oriol Fornes, Sveinung Gundersen, Morten Johansen, Eivind Hovig, Boris Lenhard, Albin Sandelin, Wyeth.W Wasserman, François Parcy, and Anthony Mathelier. JASPAR 2024: 20th anniversary of the open-access database of transcription factor binding profiles. *Nucleic Acids Research*, 52(D1):D174–D182, 2024.

[6] Walter Santana-Garcia, Jaime A Castro-Mondragon, Mónica Padilla-Gálvez, Nga Thi Thuy Nguyen, Ana Elizondo-Salas, Najla Ksouri, François Gerbes, Denis Thieffry, Pierre Vincens, Bruno Contreras-Moreira, Jacques van Helden, Morgane Thomas-Chollier, and Alejandra Medina-Rivera. RSAT 2022: regulatory sequence analysis tools. *Nucleic Acids Research*, 50(W1):W670–W676, 2022.

[7] Benjamin Jean-Marie Tremblay. universalmotif: An R package for biological motifanalysis. *Journal of Open Source Software*, 9(100):7012, 2024.

[8] Ziga Avsec, Melanie Weilert, Avanti Shrikumar, Sabrina Krueger, Amr Alexandari, Khyati Dalal, Robin Fropf, Charles McAnany, Julien Gagneur, Anshul Kundaje, and Julia Zeitlinger. Base-resolution models of transcription-factor binding reveal soft motif syntax. *Nature Genetics*, 53(3):354–366, 2021.

[9] Eric C Rouchka and C Timothy Hardin. rMotifGen: random motif generator for DNA and protein sequences. *BMC Bioinformatics*, 8(1):292, 2007.

[10] Ghadi S. Al Hajj, Johan Pensar, and Geir K. Sandve. Dagsim: Combining dag-based model structure with unconstrained data types and relations for flexible, transparent, and modularized data simulation. *PLOS ONE*, 18(4):1–9, 04 2023.

[11] Aziz Khan. pyJASPAR: a Pythonic interface to JASPAR transcription factor motifs, 2021.

[12] Aziz Khan and Anthony Mathelier. Intervene: a tool for intersection and visualization of multiple gene or genomic region sets. *BMC Bioinformatics*, 18(1):287, 2017.

[13] Avantika Lal, Laura Gunsalus, Anay Gupta, Tommaso Biancalani, and Gokcen Eraslan. Polygraph: a software framework for the systematic assessment of synthetic regulatory DNA elements. *Genome Biol.*, 26(1):114, 2025.

[14] Johannes Linder and Georg Seelig. Fast activation maximization for molecular sequence design. *BMC Bioinformatics*, 22(1):510, 2021.

[15] Sam Sinai, Richard Wang, Alexander Whatley, Stewart Slocum, Elina Locane, and Eric D. Kelsic. AdaLead: A simple and robust adaptive greedy search algorithm for sequence design, 2020. Version Number: 1.

[16] Peter J. M. Van Laarhoven and Emile H. L. Aarts. *Simulated Annealing: Theory and Applications*. Dordrecht, 1987.

[17] Eric Nguyen, Michael Poli, Marjan Faizi, Armin Thomas, Callum Birch-Sykes, Michael Wornow, Aman Patel, Clayton Rabideau, Stefano Massaroli, Yoshua Bengio, Stefano Ermon, Stephen A. Baccus, and Chris Ré. Hyenadna: Long-range genomic sequence modeling at single nucleotide resolution. *arXiv preprint arXiv:2306.15794*, 2023.

[18] Tom Whitington, Martin C. Frith, James Johnson, and Timothy L. Bailey. Inferring transcription factor complexes from ChIP-seq data. *Nucleic Acids Research*, 39(15):e98–e98, 2011.

[19] Scott Lundberg. A unified approach to interpreting model predictions. *arXiv preprint arXiv:1705.07874*, 2017.

[20] Avanti Shrikumar, Peyton Greenside, and Anshul Kundaje. Learning Important Features Through Propagating Activation Differences, 2019. arXiv:1704.02685 [cs].

[21] Damla Ovek Baydar, Ieva Rauluseviciute, Dina R Aronsen, Romain Blanc-Mathieu, Ine Bonthuis, Herman de Beukelaer, Katalin Ferenc, Alice Jegou, Vipin Kumar, Roza Berhanu Lemma, Jérémy Lucas, Mathis Pochon, Chang M Yun, Vivekanandan Ramalingam, Salil Sanjay Deshpande, Aman Patel, Georgi K Marinov, Austin T Wang, Alejandro Aguirre, Jaime A Castro-Mondragon, Damir Baranasic, Jeanne Chèneby, Sveinung Gundersen, Morten Johansen, Aziz Khan, Marieke L Kuijjer, Eivind Hovig, Boris Lenhard, Albin Sandelin, Klaas Vandepoele, Wyeth W Wasserman, François Parcy, Anshul Kundaje, and Anthony Mathelier. Jaspar 2026: expansion of transcription factor binding profiles and integration of deep learning models. *Nucleic Acids Research*, page gkaf1209, 12 2025.

[22] Jan Soroczynski, Lauren J. Anderson, Joanna L. Yeung, Justin M. Rendleman, Deena A. Oren, Hide A. Konishi, and Viviana I. Risca. OpenTn5: Open-Source Resource for Robust and Scalable Tn5 Transposase Purification and Characterization, 2024.

[23] Avanti Shrikumar, Katherine Tian, Žiga Avsec, Anna Shcherbina, Abhimanyu Banerjee, Mahfuza Sharmin, Surag Nair, and Anshul Kundaje. Technical Note on Transcription Factor Motif Discovery from Importance Scores (TF-MoDISco) version 0.5.6.5, 2018. Version Number: 5.
